# Comparative effectiveness of furosemide vs torasemide in symptomatic therapy in heart failure patients: A randomized controlled study protocol

**DOI:** 10.1097/MD.0000000000024661

**Published:** 2021-02-19

**Authors:** Yifan Li, Li Li, Zhipeng Guo, Shunye Zhang

**Affiliations:** a Department of cardiovascular surgery; b Department of critical care medicine, Shanxi Cardiovascular Hospital, Shanxi; c Department of cardiovascular surgery, TEDA International Cardiovascular Hospital, Tianjin, China.

**Keywords:** furosemide, heart failure, protocol, randomized, torasemide

## Abstract

**Background:**

We performed this randomized controlled study protocol to investigate the efficacy and adverse effects of furosemide vs torasemide in patients with heart failure (HF).

**Method:**

The present study was authorized by the local research ethics committee of Shanxi Cardiovascular Hospital (no. 48736645) and informed consent was obtained from all patients. Patients were enrolled in a consecutive prospective manner on a voluntary basis. Patients who were aged 18 years and older with HF who were eligible to enroll in this randomized trial. All patients had evidence of left ventricular systolic dysfunction, confirmed by echocardiographic or nuclear imaging. The exclusion criteria were left ventricular diastolic dysfunction only, or receipt of medical or pharmaceutical care in other health systems. The primary efficacy end point was the change in procollagen type I carboxyterminal peptide (PICP) serum levels between baseline and final visit. Secondary efficacy variables included parameters related to the clinical course of HF, such as body weight, presence of edema, signs and symptoms of HF, electrocardiogram and echocardiographic evaluation, amino-terminal pro brain-type natriuretic peptide (NT-proBNP) serum levels measured by ELISA method, systolic blood pressure (SBP), diastolic blood pressure (DBP), heart rate, and renal function.

**Results:**

One hundred patients who met the inclusion criteria were included in our study, Table 1

**Table 1:** The effects of furosemide and torsemide on measures of clinical outcomes.

	Furosemide group (n = 75)	Torsemide group (n = 75)	*P* value
PICP			
SBP			
DBP			
NT-proBNP			
Heart rate			
Body mass index			
Signs and symptoms of HF			
MLHFQ			
Complications			

showed the effects of furosemide and torsemide on measures of clinical outcomes.

**Discussion:**

Fluid overload is the primary cause of hospitalization among patients with HF. Preventing circulatory congestion requires careful control of dietary sodium and chronic administration of loop diuretics. Torasemide and furosemide are representatives of loop diuretics with an identical diuretic mechanism, but different pharmacokinetic properties and additional effects. There is a need for reliable conclusion regarding the comparison of furosemide and torasemide in patients with HF. Several limitation should be noted:

1. the small number of participants did not enable assessment of the impact of torasemide and furosemide in different clinically relevant subgroups that is, elderly, patients with chronic kidney disease, dilated cardiomyopathy;
2. short-term follow up might lead to underestimation of the complications;
3. methodological weakness in study design may affect the results. Future high quality studies were still required.

## 1 Introduction

Heart failure (HF) is a widely prevalent clinical syndrome that has a huge burden on health care systems worldwide.^[1,2]^ It approximately affects 1% to 2% of the adult population in developed countries, rising to greater than or equal to 10% of people greater than 70 years of age.^[3,4]^ Despite an intensive delivery of healthcare and education to affected patients, its incidence continues to increase, resulting in 50% or greater mortality in a 5-year observation. HF is characterized by several symptoms, including breathlessness, ankle swelling, and fatigue, and several signs such as jugular distension, pulmonary crackles, and peripheral edema.^[5,6]^ It is usually the result of structural defects and/or elevated intracardiac pressures at rest or during stress. Diuretics are recommended to reduce fluid and sodium retention in the body and relieve the symptoms of HF.^[7,8]^ Previous clinical trial has concluded that in patients with chronic HF, loop and thiazide diuretics appear to decrease the risk of death and deterioration compared to placebo, and they also appear to improve exercise in comparison with active control. Loop diuretics including torasemide and furosemide act on the thick ascending limb of the loop of Henle leading to Na-K-2Cl cotransporter inhibition leading to effective inhibition of sodium reabsorption and therefore reduction in water reabsorption.^[9]^ Furosemide is the most often used loop diuretic for HF.^[10]^ However, present data suggest potential pharmacologic and antifibrotic advantages with torsemide. The bioavailability varies between 76% to 96% and 10% to 90% for torasemide and furosemide, respectively.^[11]^ In addition, a longer duration of action and improved tolerability for torasemide over furosemide have been demonstrated in some clinical studies.^[12,13]^ However, no reliable evidence has been reached due to the poor study design and small sample size. We performed this randomized controlled study protocol to investigate the efficacy and adverse effects of furosemide vs torasemide in patients with HF.

## 2 Methods

The present study was authorized by the local research ethics committee of Shanxi Cardiovascular Hospital (no. 48736645) and informed consent was obtained from all patients. Patients were enrolled in a consecutive prospective manner on a voluntary basis. The study was registered in the public trial registry (researchregistry 6455).

Patients who were aged 18 years and older with HF who were eligible to enroll in this randomized trial. All patients had evidence of left ventricular systolic dysfunction, confirmed by echocardiographic or nuclear imaging. The exclusion criteria were left ventricular diastolic dysfunction only, or receipt of medical or pharmaceutical care in other health systems.

Following a screening period, eligible patients were randomly assigned in a ratio of 1:1 to receive torasemide (10 mg daily) or furosemide (40 mg daily), in addition to their existing HF therapy. Treatment allocation was performed in blocks, following the randomization list previously generated by a central statistician, and sequentially assigned to each center. The dose of torasemide or furosemide could be uptitrated at follow-up visits if the patient did not respond to the treatment. Maximum doses allowed were 40 mg/d for torasemide and 160 mg/d for furosemide. Patients received the assigned treatment until the end of the study at week 32 (final visit).

The primary efficacy end point was the change in procollagen type I carboxyterminal peptide (PICP) serum levels between baseline and final visit. Serum PICP was determined by specific ELISA in a central laboratory. Secondary efficacy variables included parameters related to the clinical course of HF, such as body weight, presence of edema, signs and symptoms of HF, electrocardiogram and echocardiographic evaluation, amino-terminal pro brain-type natriuretic peptide (NT-proBNP) serum levels measured by ELISA method, systolic blood pressure (SBP), diastolic blood pressure (DBP), heart rate, and renal function. Arterial BP was measured in the morning, after 10 minutes in the supine position, using a mercury column sphygmomanometer. Additionally, incidence of cardiovascular events during the follow-up period of the study was monitored. The quality of life of the patients included in the study was measured using the Minnesota Living with Heart Failure Questionnaire.

Continuous and ordinal variables were expressed as a median (interquartile range). Categorical data were presented as a number of patients and percentages. Group comparisons were performed using the Fisher exact test for qualitative variables and *t* test for quantitative, normally distributed variables, and the Mann–Whitney *U* test for quantitative, non-normally distributed variables (normality of distribution was checked with the Shapiro–Wilk test). For all analyses, a *P* value of less than .05 was considered statistically significant.

## 3 Results

One hundred fifty patients who met the inclusion criteria were included in our study, Table 1 showed the effects of furosemide and torsemide on measures of clinical outcomes.

## 4 Discussion

Fluid overload is the primary cause of hospitalization among patients with HF.^[14]^ Preventing circulatory congestion requires careful control of dietary sodium and chronic administration of oral loop diuretics.^[15,16]^ When patients with HF deteriorate, it is often presumed that they have lapsed in their adherence to diet or to use of diuretics. Poor absorption of a diuretic, however, coupled with continuous sodium intake can also cause an inexorable accumulation of sodium and water. Cardiac remodeling is an indicative of a progressive course of HF.^[17]^ Hemodynamic load and many other factors can influence the status of cardiac remodeling. Therefore, drugs that decrease the load can contribute to preventing or slowing cardiac remodeling, and this is one of the primary aims of HF therapy.

Torasemide and furosemide are representatives of loop diuretics with an identical diuretic mechanism, but different pharmacokinetic properties and additional effects. Compared to furosemide, torasemide has greater bioavailability, a higher degree of protein binding, and a longer half-life. These properties make that torasemide works faster, longer, and less frequently causes rapid micturition than furosemide. According to previous studies, torasemide decreases rates of HF hospitalizations and hospital stay, improves exercise tolerance, quality of life, left ventricular function, cardiac sympathetic nerve activity, myocardial fibrosis, pulmonary congestion, peripheral edema, and blood pressure compared with furosemide.^[12,18]^ However, the clinical evidence remains unclear. In this study, we aimed to compare clinical outcomes and adverse effects of therapy with furosemide vstorasemide in patients with HF. Several limitation should be noted:

1. the small number of participants did not enable assessment of the impact of torasemide and furosemide in different clinically relevant subgroups that is, elderly, patients with chronic kidney disease, dilated cardiomyopathy;
2. short-term follow up might lead to underestimation of the complications;
3. methodological weakness in study design may affect the results. Future high quality studies were still required.

## Author contributions

**Conceptualization:** Yifan Li.

**Data curation:** Zhipeng Guo.

**Formal analysis:** Li Li.

**Funding acquisition:** Shunye Zhang.

**Writing – original draft:** Yifan Li.
